# Biomass allocation and productivity–richness relationship across four grassland types at the Qinghai Plateau

**DOI:** 10.1002/ece3.5920

**Published:** 2019-12-11

**Authors:** Licong Dai, Xiaowei Guo, Xun Ke, Yuting Lan, Fawei Zhang, Yikang Li, Li Lin, Qian Li, Guangmin Cao, Bo Fan, Dawen Qian, Huakun Zhou, Yangong Du

**Affiliations:** ^1^ Qinghai Provincial Key Laboratory of Restoration Ecology for Cold Region Northwest Institute of Plateau Biology Chinese Academy of Sciences Xining China; ^2^ Key Laboratory of Adaptation and Evolution of Plateau Biota Northwest Institute of Plateau Biology Chinese Academy of Sciences Xining China; ^3^ College of Life Sciences Luoyang Normal University Luoyang China; ^4^ University of Chinese Academy of Science Beijing China

**Keywords:** above‐ and belowground biomass allocation, grassland types, productivity–richness relationship, Qinghai Plateau, reduced major axis

## Abstract

Aboveground biomass (AGB) and belowground biomass (BGB) allocation and productivity–richness relationship are controversial. Here, we assessed AGB and BGB allocation and the productivity–richness relationship at community level across four grassland types based on the biomass data collected from 80 sites across the Qinghai Plateau during 2011–2012. The reduced major axis regression and general linear models were used and showed that (a) the median values of AGB were significantly higher in alpine meadow than in other three grassland types; the ratio of root to shoot (R/S) was significantly higher in desert grassland (36.06) than intemperate grassland (16.60), alpine meadow (13.35), and meadow steppe (19.46). The temperate grassland had deeper root distribution than the other three grasslands, with about 91.45% roots distributed in the top 30 cm soil layer. (b) The slopes between log AGB and log BGB in the temperate grassland and meadow steppe were 1.09 and 1, respectively, whereas that in the desert grassland was 1.12, which was significantly different from the isometric allocation relationship. A competitive relationship between AGB and BGB was observed in the alpine meadow with a slope of −1.83, indicating a trade‐off between AGB and BGB in the alpine meadow. (c) A positive productivity–richness relationship existed across the four grassland types, suggesting that the positive productivity–richness relationship might not be affected by the environmental factors at the plant location. Our results provide a new insight for biomass allocation and biodiversity–ecosystem functioning research.

## Graphical abstract

The median values of aboveground biomass (AGB) were significantly higher in alpine meadow than in other three grassland types. The temperate grassland had deeper root distribution than the other three grasslands, with about 91.45% roots distributed in the top 30 cm soil layer. A positive productivity–richness relationship existed across the four grassland types.

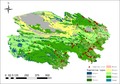

## 1 INTRODUCTION

Aboveground biomass (AGB) and belowground biomass (BGB) allocation and productivity–richness relationship are majorly studied in plant ecology (Fraser et al., 2015; Yang, Fang, Ma, Guo, & Mohammat, 2010). The AGB and BGB allocation not only affects the performance of the individual plant, but also alters its structure and reproductive functions, thereby affecting the carbon cycle of the terrestrial ecosystem (Mokany, Raison, & Prokushkin, 2006). To date, two hypotheses were proposed in biomass allocation pattern, that is, optimal partitioning and isometric allocation (McCarthy & Enquist, 2007; Shipley & Meziane, 2002; Zeng, Wu, & Zhang, 2015). The optimal partitioning hypothesis indicates that the plants tend to allocate more biomass to the organ with restricted growth to in order to obtain more resource (McCarthy & Enquist, 2007; Shipley & Meziane, 2002; Zeng et al., 2015), and such allocation pattern was shaped by external environmental conditions such as climate and nutrient conditions (McCarthy & Enquist, 2007). For instance, plants tend to allocate more biomass to roots when water or nutrients are the limiting factor, and allocate more biomass to shoots when light is limiting (Crist & Stout, 1929; Hunt & Burnett, 1973). The isometric allocation hypothesis suggested that the BGB increase along with the increase in AGB in an isometric manner, that is, the slope value of log AGB and log BGB did not significantly differ from 1.0, and this relationship remained unaffected by the environmental conditions such as nitrogen levels and water conditions (Yang, Fang, Ji, & Han, 2009). Although AGB and BGB allocation pattern have been widely explored in the recent years, the tactics of biomass allocation in plants remain controversial (Enquist & Niklas, 2002; McCarthy & Enquist, 2007), with the generality of isometric allocation and optimal partitioning theory under question. For example, broad‐scale biomass observations revealed that the AGB and BGB allocation of vascular plants followed isometric allocation (Enquist & Niklas, 2002); however, few manipulative experiments did not support this hypothesis (McConnaughay & Coleman, 1999; Shipley & Meziane, 2002). Considering that the AGB and BGB biomass allocation pattern may vary with the grassland types, previous studies have emphasized the individual level or one grassland type and emphasis on the different grassland types at the community level was scarce (McConnaughay & Coleman, 1999). For instance, one study indicated that the AGB and BGB allocation conform to the isometric relationship at the level of individual plants (Cheng & Niklas, 2006); however, whether this relationship exists at the community level remains unclear, in particular, in different grassland types. Thus, there is urgent to examine the relationship among various grasslands at the community level. Furthermore, the resource spatial gradient at the community location should be also considered for exploring the AGB and BGB allocation (Ma et al., 2013).

In addition, the relationship between productivity and species richness was also an important central issue in plant ecology for decades (Fraser et al., 2015; Waide et al., 1999). Nevertheless, no consensus has been obtained regarding the relationship between productivity and species richness along natural gradients (Tredennick et al., 2016). Three dominant views were put forth regarding the productivity–richness relationship in terrestrial ecosystems, that is, hump‐shaped or U shaped (Ni, Wang, Bai, & Li, 2007; Waide et al., 1999), positive relationship (Bai et al., 2007; Marquard et al., 2009), and no correlation (Gao, Men, & Ge, 2014). The hump‐shape indicated that plant diversity reach its highest value at intermediate levels of productivity owing to density limitation (Oksanen, 1996), dispersal limitation, and evolutionary history (Zobel & Pärtel, 2008). The positive relationship indicated that the plant species richness increased with increasing productivity because of the separating effects of niche partitioning and complementarity effects (Fargione et al., 2007; Marquard et al., 2009). Previous meta‐analysis from 171 published studies suggested that the hump‐shaped curve was the dominated form of productivity–richness relationship both at local and regional scales (Mittelbach et al., 2001). A saturating curve is commonly found in previous study that the aboveground biomass reach its highest value at intermediate levels of species richness (Tilman et al., 2001). Besides, the relationship between productivity and species richness was also shaped by spatial scale. For instance, the relative effects of species richness tend to be highest at small‐to‐intermediate spatial scales but decreased at regional scales owing to its greater environment heterogeneity (Loreau et al., 2001). Furthermore, the grassland types may also an important factor in altering the shape of relationship between productivity and species richness because of its different species composition and structure (Fraser et al., 2015). Combine those, the grassland types and spatial scale should be considered in the productivity–richness relationship. Nevertheless, a majority of previous studies explored the productivity–richness relationship mainly limited by one grassland type or local scale (Wang, Niu, Yang, & Zhou, 2010), with little attention on the productivity–richness relationship in different biomes at a large spatial scale (Zhang et al., 2011). Therefore, it is necessary to assess the universality of productivity–richness relationship in different biomes at a large spatial scale to provide a new sight for the prediction of plant diversity in grasslands.

In the present study, using the data surveyed from 80 sites across the Qinghai plateau, we aimed to (a) explore biomass allocation and the relationship between productivity and species richness across four grasslands (temperate grassland, desert grassland, alpine meadow, and meadow steppe) with different climate, (b) examine the vertical distribution of roots across four grassland types. We hypothesized the biomass allocation between aboveground biomass and belowground biomass may vary with grassland types.

## 2 MATERIALS AND METHODS

### 2.1 Study area

**Figure 1:**
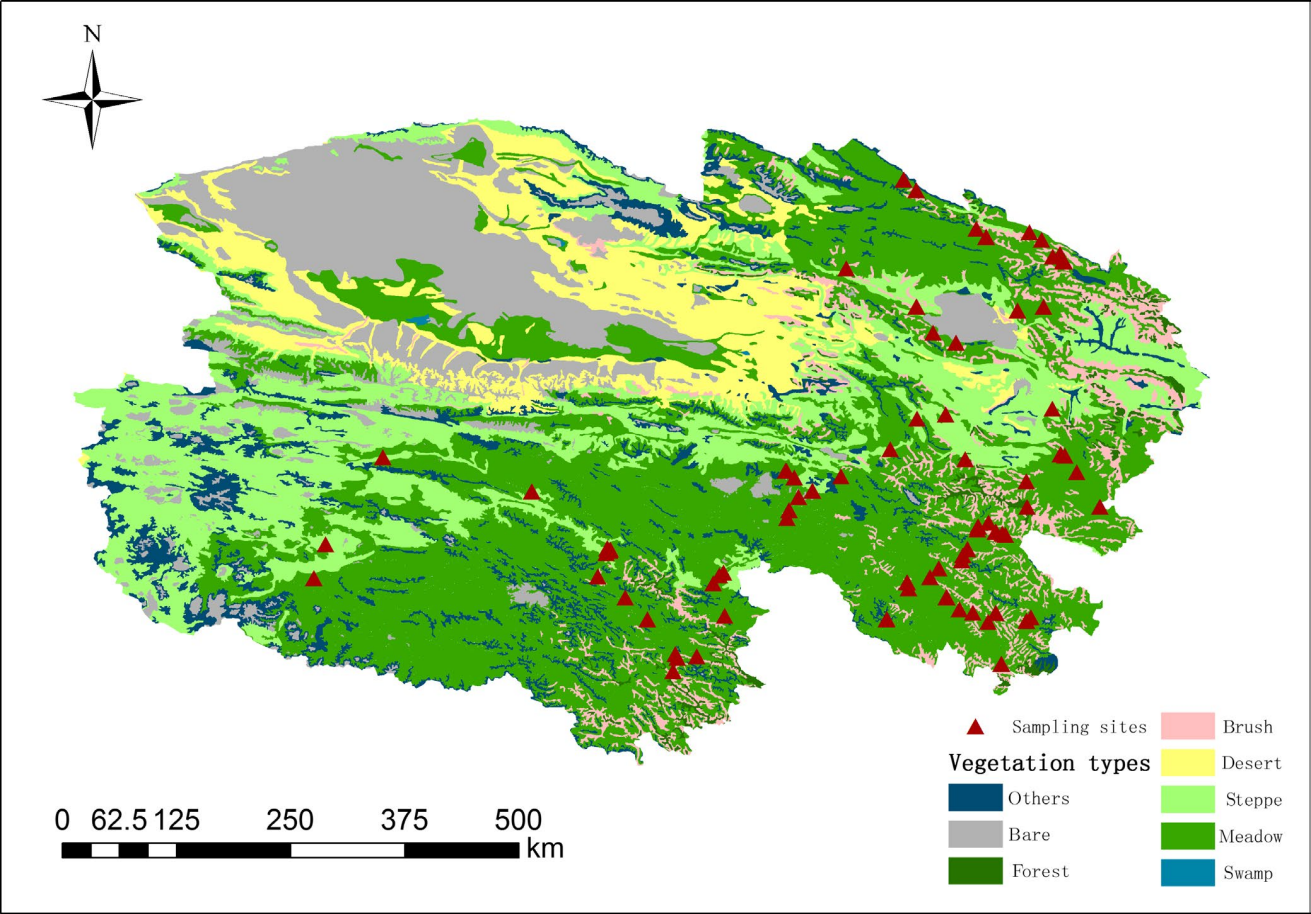
The spatial distribution of sampling sites on the Qinghai Plateau, the locations of sampling sites surveyed are displayed on the vegetation map based on a scale of 1:1,000,000 (Chinese Academy of Sciences, 2001) during 2011–2012 (Liu et al., 2016)

This study was conducted on the Qinghai–Tibetan Plateau in Qinghai Province, which covered an area of 36.37 × 10^4^ km^2^, average elevation of 4,000 m, longitude range from 92.17 to 101.75°E, latitude range from 30.29 to 38.60°N, mean annual precipitation range from 400 to 800 mm (with 80% of precipitation occurring in summer season), and the annual air temperature range from −5 to 12°C. In total, four grassland types are observed across 80 sampling sites based on China's vegetation classification system, that is temperate grassland, desert grassland, alpine meadow, and steppe meadow (locations are presented in Figure 1). Steppe and alpine meadows were characterized by frozen soil and permafrost. The detail information about the dominant vegetation and distribution area in each grassland type was found in Table 1 that extract from previous study in the same study site (Liu et al., 2016).

**Table 1:** The characteristics of plants and distribution across four grasslands type in Qinghai Province

Grassland types	Dominant vegetation	Distribution
Temperate grassland	*Achnatherum splendens* (Trin.) Nevski, *Agropyron cristatum *(L.) Gaertn, Subgen.* Oxytropis ochrocephala* Bunge, *Aster tataricus* L. f. Coverage is approximately 65%–80%	Near Qinghai Lake
Alpine meadow	*Kobresia pygmaea* C. B. Clarke. Coverage is approximately 50%–70%	South Qinghai Province
Desert grassland	*Ceratoides latens* (J. F. Gmel.) *Reveal et Holmgren* and *Kalidium foliatum* (Pall.) Moq..Coverage is low	NW Qinghai Province
Meadow steppe	*Kobresia humilis* (C. A. Mey. ex Trautv.) Sergiev and *Leontopodium leontopodioides* (Willd.) Beauv.; coverage is approximately 50%	NE Qinghai Province

### 2.2 Data collection

We harvested the AGB and BGB across 80 sites on the Qinghai–Tibetan Plateau once during summer (July to September) in 2011 and 2012. Five quadrats of 0.25 m^2^ (0.5 × 0.5 m) were sampled in the alpine meadow at each site (100 × 10 m) due to the abundant AGB in alpine meadow, and five quadrats of 1 m^2^ (1 × 1 m) were sampled in the temperate grassland, desert grassland, and meadow steppe at each site. The AGB was measured via the clipping method at each quadrats at 20 m intervals along the 100 × 10 m site across the four grassland types, whereas the BGB was sampled from the core of the soil (diameter, 6 cm) within each quadrat at depths of 0–5, 5–10, 10–20, 20–30, 30–50, and 50–70 cm. Five soil samples from each depth interval on the same quadrats were combined, followed by root cleaning and removal of the soil particles, and the live and dead roots were not distinguished, thus the BGB include both live and dead. Eventually, both AGB and BGB samples were oven‐dried at 65°C to a constant weight. In this study, the root‐to‐shoot ratio (R/S) was calculated as the ratio of BGB to AGB, the AGB was sampled at its peak time, which approximated considered as the aboveground net primary productivity, and the species richness was estimated by the total number of plant species in each quadrat.

### 2.3 Data analysis

First, the biomass data were subjected to preliminary normality test in order to assess the normality of the data; thereafter, the median values of AGB, BGB, and R/S ratio were calculated across 80 study sites. Furthermore, we classified all sites into temperate grassland, desert grassland, alpine meadow, and meadow steppe based on a vegetation map at a scale of 1:1,000,000 (Chinese Academy of Sciences, 2001), and the overall AGB, BGB, and R/S ratio for the four grasslands were calculated. Given we have no ideal about which variable is *X* and which is *Y*, then we explored the relationship between log AGB and log BGB using reduced major axis (RMA) regression instead of ordinary least squares (OLS), because RMA is symmetric, indicated that a single line defines the bivariate relationship, no need to consider which variable is *X* and which is *Y* (Smith, 2009). Whereas the OLS is asymmetric, lead to the slope and resulting interpretation of the data are different when the variables assigned to *X* and *Y* are reversed (Smith, 2009). The software package “Standardized Major Axis Tests and Routines” was applied to examine the slope (*a*) and *y*‐intercept (log *b*) of the allocation function (Falster, Warton, & Wright, 2003). We examined the relationship between AGB and species richness by general linear model (GLM).

The vertical distribution of roots was characterized using the asymptotic function by Gale and Grigal (1987), as follows:

$$Y=1-\beta^{d}$$

where *β* is the estimated parameter, *Y* is the cumulative percentage of root biomass from the soil surface to depth *d* (cm); the values of *β* range from 0 to 1, and larger *β* value indicates deeper root distribution. All statistical analysis was performed in R (R Development Core Team, 2006).

## 3 RESULTS

### 3.1 Size of AGB, BGB, and R/S across four grassland types

The AGB, BGB, and R/S exhibited large variations among the four grassland types (Table 2). In temperate grassland, ranging from 23.25 to 172.39 g/m^2^ for AGB, 1,189.44–4,516.28 g/m^2^ for BGB and 7.49–70.64 for R/S values (Table 2); in the desert grassland, ranging from 3.25 to 54.37 g/m^2^ for AGB, 180.88–1,650.78 g/m^2^ for BGB, and 9.23–69.10 for R/S (Table 2); in the alpine meadow, ranging from 29.77 to 399.56 g/m^2^ for AGB, 141.63–6,344.11 g/m^2^ for BGB, and 1.72–66.56 for R/S (Table 2); and in the meadow steppe, ranging from 24.55 to 232.33 g/m^2^ for AGB, 116.65–8,370.78 g/m^2^ for BGB, and 3.20–39.97 for R/S (Table 2). Overall, the median value of AGB in the alpine meadow (137.29 g/m^2^) was significantly higher than that in the temperate grassland (85.59 g/m^2^), desert grassland (10.06 g/m^2^), and meadow steppe (96.34 g/m^2^) (Table 2). However, the median value of BGB in the meadow steppe was significantly higher than that in the other three grassland types; whereas the R/S in the desert grassland (36.06) was significantly higher than that of the temperate grassland (16.60), alpine meadow (13.35), and meadow steppe (19.46) (Table 2).

**Table 2:** Median values of aboveground biomass (AGB), belowground biomass (BGB), and root: shoot (R:S) ratio for temperate grassland, desert grassland, alpine meadow, and meadow steppe

Grassland type	AGB (g/m^2^)		BGB (g/m^2^)		R:S ratio		*n*
	Median	Range	Median	Range	Median	Range	
Temperate grassland	85.59^b^	23.25–172.39	1,534.77^c^	1,189.44–4,516.28	16.60^b^	7.49–70.64	16
Desert grassland	10.06^c^	3.25–54.37	761.05^d^	180.88–1,650.78	36.06^a^	9.23–69.10	11
Alpine meadow	137.29^a^	29.77–399.56	2,637.98^b^	141.63–6,344.11	13.35^c^	1.72–66.56	23
Meadow steppe	96.34^b^	24.55–232.33	3,319.22^a^	116.65–8,370.78	19.46^b^	3.20–39.97	30

Note

Different letters indicate significant differences between two grassland types at 0.05 level.

### 3.2 Vertical distribution of roots across four grassland types

**Figure 2:**
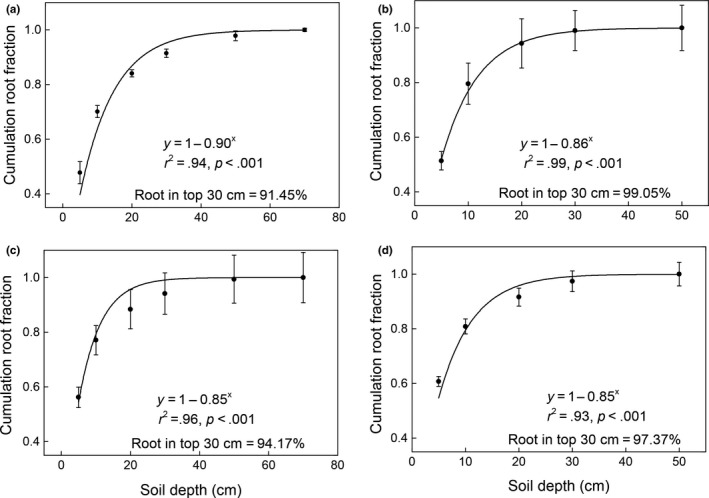
Vertical distributions of roots in (a) temperate grassland, (b) desert grassland, (c) meadow steppe, and (d) alpine meadow. The vertical distribution of roots across four grassland types was fitted by the function proposed by Gale and Grigal (1987)

**Figure 3:**
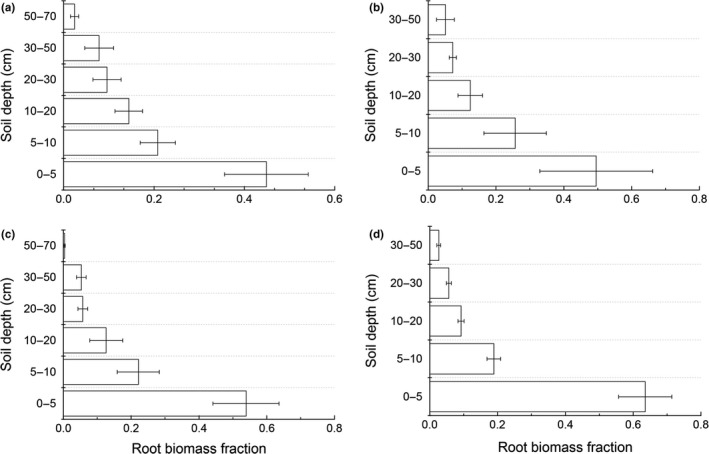
Root biomass fraction across different soil layers in (a) temperate grassland, (b) desert grassland, (c) meadow steppe, and (d) alpine meadow

There was great variation about vertical distribution of roots across four grassland types, more root biomass was observed in the top soil layer for desert grassland compared with other three vegetation types (Figure 2), with 99.05% of roots in the top 30 cm of soil for desert grassland (*β* = .86, *r* ^2^ = .99, *p* < .001; Figure 2b) compared to 91.45% for temperate grassland (*β* = .90, *r* ^2^ = .94, *p* < .001; Figure 2a), 94.17% for meadow steppe (*β* = .85, *r* ^2^ = .96, *p* < .001; Figure 2c), and 97.37% for alpine meadow (*β* = .85, *r* ^2^ = .93, *p* < .001; Figure 2d). Moreover, the 0–5 cm root fraction of the temperate grassland was 0.45, which was smaller than that of the desert grassland (0.50), alpine meadow (0.64) and meadow steppe (0.54) (Figure 3). This indicated that the temperate grassland has a deeper root distribution than the other three grasslands.

### 3.3 AGB and BGB allocation across the four grassland types

**Figure 4:**
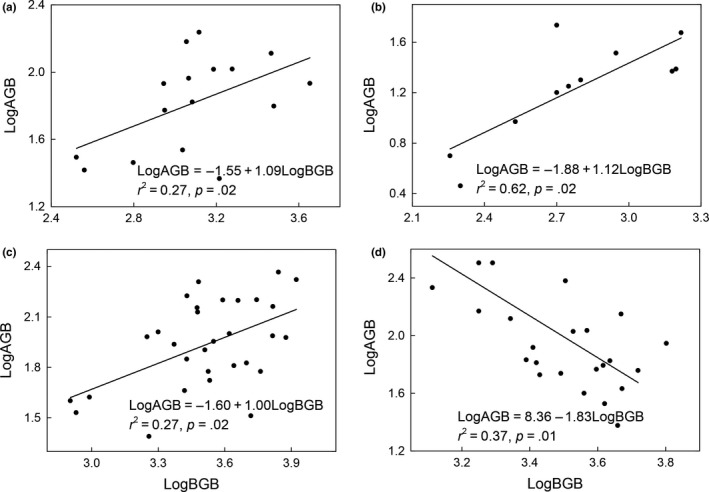
Relationships between aboveground biomass (AGB) and belowground biomass (BGB) in (a) temperate grassland, (b) desert grassland, (c) meadow steppe, and (d) alpine meadow

The slope (*a*) of the relationship between log AGB and log BGB in the temperate grassland and meadow steppe was 1.09 and 1, respectively (Figure 4a,c), which did not differ significantly from the isometric relationships; whereas the slope (*a*) of the relationship between log AGB and log BGB in the desert grassland was 1.12 (Figure 4b), which differed significantly from the isometric relationships (i.e., slope = 1), and supported the allometric relationship. Interestingly, the slope (*a*) of the relationship between log AGB and log BGB in the alpine meadow was −1.83 (Figure 4d), which indicated there may a competitive relationship between AGB and BGB in the alpine meadow.

### 3.4 Relationship between productivity and species richness

**Figure 5:**
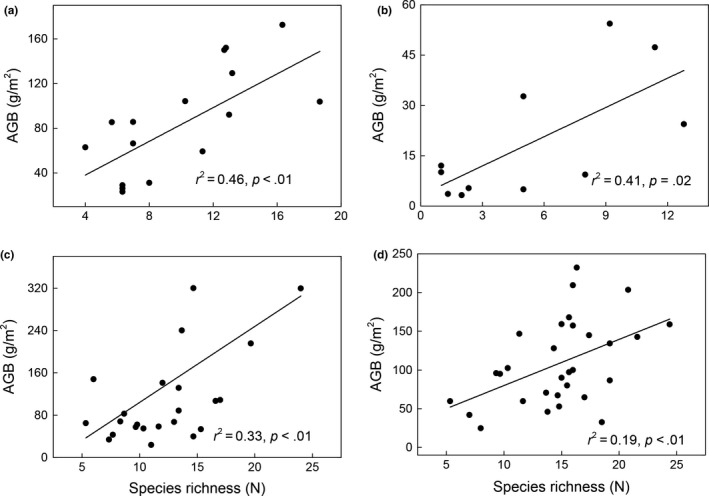
Relationships between aboveground biomass (AGB) and species richness in (a) temperate grassland, (b) desert grassland, (c) meadow steppe, and (d) alpine meadow

**Figure 6:**
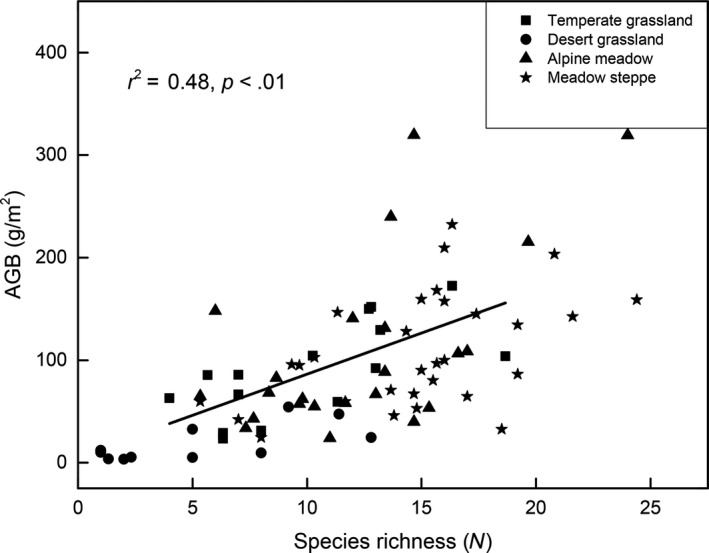
Relationships between aboveground biomass (AGB) and species richness across four grassland types

The general linear model (GLM) indicated that a significant positive relationship existed between the species richness and AGB across the four grasslands (*p* < .01; Figure 5). To further confirm the positive relationship between the species richness and AGB, we analyzed the productivity–richness relationship by compiling the data of the four grasslands, wherein our result revealed a significant positive relationship between the species richness and AGB (Figure 6).

## 4 DISCUSSION

### 4.1 R/S and vertical distribution of roots across four grassland types

Our result indicated that the desert grassland exhibited maximum R/S value, whereas the alpine meadow revealed minimum R/S value among the four grasslands, which was consistent with the previous observations at species level (Wang et al., 2010). A possible explanation for the difference in the R/S values among the different grassland types might be attributed to the difference in climate at the plant community location. For instance, the plant in the desert grassland faced water scarcity. In general, the plant allocated more biomass to the roots in low‐moisture and poor nutrient conditions, and more biomass to the shoot when the nutrient and moisture was abundant, according to the optimum allocation hypothesis (McConnaughay & Coleman, 1999).Thus, the plant in the desert grassland might preferably allocate more biomass into the roots in order to capture more soil moisture to ease water stress; this is supported by the allometric allocation relationship in the desert grassland (slope = 1.12). Furthermore, we found that the R/S in our study were significantly higher than that reported by previous study across China's grasslands at community level (Yang et al., 2010). A potential explanation for the differences in R/S between our study and previous studies at community levels was difficulties in identifying dead or live roots (Wang et al., 2010). In our study, the root biomass might be overestimated owing to the difficulty to separate dead roots from living ones just by color and consistency (Wang et al., 2016). Thus intensive and systematic studies are needed for both the individual and community levels at the same study site in the future.

Furthermore, we observed less root biomass in the top 30 cm soil layer in the temperate grasslands (91.45) than the desert grassland (99.05%), alpine meadow (97.37%), and meadow steppe (94.17%), indicating that the temperate grasslands have deeper root distribution compared with other three grassland types, which was in accordance with a previous study (Jackson et al., 1996). Meanwhile, previous study reported that the vertical distribution of roots was affected by several factors such as soil texture, shoot biomass, and soil nutrients (Yang et al., 2009). For instance, the alpine meadow and meadow steppe had widely distributed permafrost or seasonally frozen ground, which may inhibit the root growth to deep soil layers in cold regions (Jackson et al., 1996). Therefore, a detailed research on both soil and climatic conditions should be considered in future studies.

### 4.2 Above‐ and belowground biomass allocation across four grassland types

Several studies reported that the isometric relationship widely exists across both grassland and forest ecosystems and is independent of the environmental factors (Cheng & Niklas, 2006; Fang, Oikawa, Kato, Mo, & Wang, 2005), indicating the generality of the relationships between AGB and BGB across different biomes. Nevertheless, this general pattern of isometric relationship was not supported by our results. Our results reported that isometric relationship only exists in temperate grassland, whereas the desert grassland followed allometric relationship, indicating that the biomass allocation vary with the grassland types, which support our initial hypothesis. Furthermore, a competitive relationship was observed in the alpine meadow between AGB and BGB, which was not in accordance with the previous study, which reported that the alpine grassland followed an isometric relationship (Yang et al., 2009). Thus, we conclude that the variation in the biomass allocation reflects a balancing strategy among the community to due to the differences in the traits of the species occupying those grasslands or differences within species in how they respond to different environmental conditions. For instance, in the desert ecosystem, the plant in low precipitation condition could allocate less biomass to the leaf to avoid more moisture loss via evapotranspiration and allocate more biomass to the root to obtain more moisture and soil nutrients from a greater volume of soil (Fan et al., 2009; Wu et al., 2013). However, in the alpine ecosystem with low temperature and short growth season, the nutrient content was less as the soil nutrient supplement of the alpine ecosystems is strongly determined by nutrient mineralization (Dai, Ke, Du, Zhang, et al., 2019; Dai, Ke, Guo, et al., 2019).Therefore, there may exist trade‐off between belowground organ to capture soil nutrient and aboveground organ to capture more carbon, according to the carbon is fixed by leaves and the water or mineral nutrients were absorbed by root (McConnaughay & Coleman, 1999); that is, the plant might choose to obtain more biomass to plant organs that associated with the acquisition of that resource at the cost of reducing the structures biomass associated with acquisition of another resource. Moreover, this evidence was supported by the seasonal dynamics of AGB and BGB in the alpine ecosystem in a previous study (Dai, Guo, Du, et al., 2019). For instance, as the solar rays are stronger from June to August, the plant could preferentially allocate more biomass to leaves to capture more carbon and reduce the root biomass that captures more soil nutrient, whereas the alpine plants tend to allocate more biomass to the root during the late growth period to in order to capture more soil nutrient at the expenses of decreasing light capture (Shipley & Meziane, 2002). This unique trade‐off might reflect an ecological strategy of the alpine plants responding to the extreme environmental conditions at the alpine ecosystem.

### 4.3 Relationship between productivity and species richness

The relationship between productivity and species richness is a central issue in plant ecology since decades, as a previous study reported that a unimodal relationship is observed worldwide (Fraser et al., 2015). Nevertheless, our results indicated that the positive linear relationship between productivity and species richness was widely observed across the four grasslands (Figure 5) and our reanalysis after compiling the data for the four grasslands, display a positive linear relationship between the productivity and species richness (Figure 6), suggesting that the positive linear productivity–richness relationship may be dominant at the Qinghai Plateau. This result was in accordance with a study by Marquard et al. (2009) who reported that the positive relationship predominates the productivity–richness relationship due to diversity‐induced changes in the size or density of individual plants; however, it is not consistent with the worldwide evidence that the terrestrial ecosystems are majorly dominated by the hump‐shaped form of productivity–richness relationship (Fraser et al., 2015). Those discrepancies may associate with type of grassland considered, range of aboveground biomass production, and the length of the gradient sampled (Fraser, Jentsch, & Sternberg, 2014; Whittaker, 2010). Firstly, the range of aboveground biomass production in our study was relative narrow (only range from 3.25 to 399.56 g/m^2^), the maximum aboveground biomass in our study may less the productivity intermediate of actual maximum aboveground biomass. Secondly, the aboveground biomass in our study not include plant litter, while there are increasing evidences indicated that the litter production is a vital part of annual net primary productivity in grasslands and have significant impact on the functioning and structure of communities by altering nutrient cycling (Knapp & Seastedt, 1986). In fact, the hump‐shaped was originally defined in terms of live plus litter material (Grime, 1973). Furthermore, the spatial scale may be an alternative explanation for these discrepancies. For example, Mittelbach et al. (2001) reported that the productivity–richness relationship varied with the spatial scale, wherein the unimodal relationships between the species diversity and productivity are more likely to occur at smaller spatial or organizational scales, the positive linear relationship is more likely occur at larger scale. Bond and Chase (2002) also reported that the hump‐shaped relationship between diversity and productivity occurs at local level, but a positively linear relationship occurs at regional level owing to the regional complementarity and sampling effect. Given the sampling area in our study was relative large, which spanned a variety of grassland community types and a broad range of climatic zones, with mean annual precipitation ranged from 400 to 800 mm, and the annual air temperature ranged from −5 to 12°C, which displayed a wide heterogeneous environment. As different species reveal diverse utilization of the environmental resources, the increased species richness enables use of maximal resources at both time and space scales, subsequently leading to a higher productivity. Furthermore, the sampling effects indicate that the complex systems with multiple species are more likely to comprise high‐yielding species than simple systems with small species (Hector, 1998; Huston, 1997). Nevertheless, as a majority of studies assessing the relationship between functional diversity and productivity mainly focus on the small scale and homogeneous habitat, the dimensions and spatial heterogeneity are often neglected (Bai et al., 2012; Zhang et al., 2011), which may result in different results between our study and previous studies considering the productivity–richness relationship.

Furthermore, certain studies have reported that the productivity–richness relationship of natural ecosystems was affected by the resource supplement available at the plant location (Ma et al., 2013). He, Bazzaz, and Schmid (2002) reported that a significant positive productivity–richness relationship was only observed at the high nutrient level, and not at the low nutrient level; however, this evidence was not observed in our study. In our study, the significant positive productivity–richness relationship existed extensively across the four grassland types both at lower and higher nutrient levels, suggesting that the positive productivity–richness relationship may not be affected by the soil resource. Considering that our study was based only on 80 study sites, the sample sizes might be too small to capture all the characteristics of the productivity–richness relationship. Accordingly, more study sites are required to have a better understanding regarding the productivity–richness relationship in the future study.

## 5 CONCLUSIONS

Considering high uncertainty in the AGB and BGB allocation and productivity–richness relationship, this study was designed to explore the AGB and BGB allocation and productivity–richness relationship across the four grassland types at the Qinghai Plateau, wherein we found that the biomass allocation pattern varied with the grassland types, wherein the temperate grassland and meadow steppe followed the isometric relationship, whereas the desert grassland followed the allometric relationship. Most importantly, a competitive relationship was observed between the AGB and BGB in the alpine meadow, reflecting a trade‐off between the aboveground and underground parts in the alpine plant. In addition, the positive productivity–richness relationship was widely observed in the four grassland types, suggesting the generality of the positive productivity–richness relationship at the Qinghai Plateau.

## CONFLICT OF INTEREST

The authors declare no conflict of interest.

## AUTHOR CONTRIBUTIONS

L Dai and X Guo performed the research, analyzed data, and wrote the paper; Y Lan, B Fan, D Qian, F Zhang, H Zhou, X Ke, L Lin, Q Li, Y Li, and Y Du analyzed data; G Cao conceived the study.

## Data Availability

The biomass and species richness data are available in Dryad: https://doi.org/10.5061/dryad.j9kd51c7s.
